# Evolutionary genomics of mycovirus-related dsRNA viruses reveals cross-family horizontal gene transfer and evolution of diverse viral lineages

**DOI:** 10.1186/1471-2148-12-91

**Published:** 2012-06-20

**Authors:** Huiquan Liu, Yanping Fu, Jiatao Xie, Jiasen Cheng, Said A Ghabrial, Guoqing Li, Youliang Peng, Xianhong Yi, Daohong Jiang

**Affiliations:** 1 State Key Laboratory of Agricultural Microbiology, Huazhong Agricultural University, Wuhan, 430070, Hubei Province, People’s Republic of China; 2 The Provincial Key Lab of Plant Pathology of Hubei Province, College of Plant Science and Technology, Huazhong Agricultural University, Wuhan, 430070, Hubei Province, People’s Republic of China; 3 Department of Plant Pathology, University of Kentucky, 201 F Plant Science Building, 1405 Veterans Drive, Lexington, KY, 40546-0312, USA; 4 State Key Laboratories for Agrobiotechnology, China Agricultural University, Yuanming-Yuan West Road No. 2, Haidian District, 100193, Beijing, People’s Republic of China; 5 Purdue-NWAFU Joint Research Center, Northwest A&F University, Yangling, 712100, Shaanxi Province, People’s Republic of China

## Abstract

**Background:**

Double-stranded (ds) RNA fungal viruses are typically isometric single-shelled particles that are classified into three families, *Totiviridae*, *Partitiviridae* and *Chrysoviridae*, the members of which possess monopartite, bipartite and quadripartite genomes, respectively. Recent findings revealed that mycovirus-related dsRNA viruses are more diverse than previously recognized. Although an increasing number of viral complete genomic sequences have become available, the evolution of these diverse dsRNA viruses remains to be clarified. This is particularly so since there is little evidence for horizontal gene transfer (HGT) among dsRNA viruses.

**Results:**

In this study, we report the molecular properties of two novel dsRNA mycoviruses that were isolated from a field strain of *Sclerotinia sclerotiorum*, Sunf-M: one is a large monopartite virus representing a distinct evolutionary lineage of dsRNA viruses; the other is a new member of the family *Partitiviridae.* Comprehensive phylogenetic analysis and genome comparison revealed that there are at least ten monopartite, three bipartite, one tripartite and three quadripartite lineages in the known dsRNA mycoviruses and that the multipartite lineages have possibly evolved from different monopartite dsRNA viruses. Moreover, we found that homologs of the S7 Domain, characteristic of members of the genus *phytoreovirus* in family *Reoviridae* are widely distributed in diverse dsRNA viral lineages, including chrysoviruses, endornaviruses and some unclassified dsRNA mycoviruses. We further provided evidence that multiple HGT events may have occurred among these dsRNA viruses from different families.

**Conclusions:**

Our study provides an insight into the phylogeny and evolution of mycovirus-related dsRNA viruses and reveals that the occurrence of HGT between different virus species and the development of multipartite genomes during evolution are important macroevolutionary mechanisms in dsRNA viruses.

## Background

Mycoviruses (fungal viruses) are widespread in all major fungal groups and most of these cause little or no obvious symptoms in their fungal hosts. Current studies on mycoviruses are mainly focused on those of economically important fungi that are typically phytopathogenic fungi. These studies aim to develop mycoviruses as biocontrol agents to combat fungal diseases and exploit them as tools to explore the physiology of their fungal hosts [1,2]. The recent discoveries of two novel mycoviruses from the white mold fungus *Sclerotinia sclerotiorum*, one RNA virus related to rubi-like viruses of positive-strand RNA viruses [3] and another circular single-stranded (ss) DNA virus related to plant geminiviruses [4], have potentially significant implications for understanding the origin and evolution of related viral lineages. Given that mycoviruses represent essential evolutionary lineage of viruses from one of three kingdoms (plants, fungi and animals), the discovery of novel mycoviruses may not only expand our knowledge of viral diversity and global ecology, but also helps shed light on the evolutionary relationships of viruses.

The majority of known mycoviruses has rigid particles and double-stranded (ds) RNA genomes and are now classified, based on the number of genome segments, into four families: *Chrysoviridae*, *Partitiviridae*, *Reoviridae*, and *Totiviridae *[5]. Members of family *Totiviridae* have monopartite dsRNA genomes coding for a capsid protein (CP) and an RNA-dependent RNA polymerase (RdRp). The genomes of members in the families, *Partitiviridae*, *Chrysoviridae* and *Reoviridae* comprise two, four and eleven/twelve segments, respectively. In addition, viruses in the family *Endornaviridae*, which lack true virions, are currently classified as dsRNA viruses by ICTV, although there is some evidence to suggest that they have evolved from alpha-like viruses of positive strand RNA viruses. Beside fungi, viruses in these families also infect other organisms.

More recently, several monopartite dsRNA viruses with evolutionary links between totiviruses and partitiviruses were isolated from plants [6-8]. Furthermore, a novel bipartite dsRNA mycovirus phylogenetically distantly related to totiviruses and chrysoviruses was reported from the white root rot fungus *Rosellinia necatrix *[9]. These viruses represent new evolutionary lineages of dsRNA viruses, implying that dsRNA viruses are more diverse than previously recognized. Although an increasing number of complete genomic sequences of viruses have been reported, the evolution of these diverse dsRNA viruses remains to be clarified.

Horizontal gene transfer (HGT) between different viruses is important in virus evolution. The occurrence of HGT is dependent on recombination or reassortment and is capable of generating impressive genetic diversity. While HGT is commonly found in retroviruses, DNA viruses and positive-sense RNA viruses [10-16], it has only rarely been shown to occur in negative-sense RNA viruses and dsRNA viruses [17,18]. In dsRNA viruses, there are only some sporadic reports of intra-species HGT events that occur in rotaviruses [19-21] and birnaviruses [18]. To date no evidence has been reported of inter-species HGT in dsRNA viruses.

Here, we carried out molecular cloning and complete genomic sequencing of two novel dsRNA viruses from *S. sclerotiorum* strain Sunf-M, one is monopartite and the other is bipartite. We then performed genome sequence comparisons and phylogenetic analyses involving these two viruses and other related known dsRNA viruses in order to elucidate the phylogenetic relationships and evolution of relevant dsRNA viruses. Moreover, we provided evidence based on sequence comparison and phylogenetic analysis that cross-family HGT events may have occurred between dsRNA viruses from different families.

## Results and discussion

### Discovery and complete genomic sequencing of dsRNA in *S. Sclerotiorum* sunf-M

**Figure 1:**
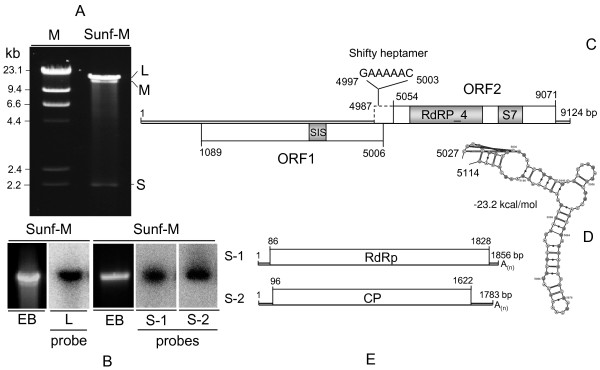
**Molecular characteristics of L- and S-dsRNA in*S. sclerotiorum*strain Sunf-M.** (**A**) Agarose gel electrophoresis of dsRNA isolated from mycelial extracts of Sunf-M. The nucleic acid preparation was fractionated on 1.0% agarose gel and stained with ethidium bromide. Lane M, DNA size markers generated by digestion of λDNA with HindIII. (**B**) Northern hybridization analysis of L- and S-dsRNA. dsRNAs were separated on a 1.0% agarose gel, transferred on to Hybond-N + membrane and hybridized with ^32^P-labelled probes prepared by random-primer labelling of cloned cDNA to L, S-1 and S-2 dsRNA, respectively. (**C**) Schematic representation of the genomic organization of L-dsRNA shows the presence of two ORFs. The dotted line box indicates a possible extension of ORF2 by frameshifting. The conserved domains of deduced proteins are shown: SIS, Sugar ISomerase domain; S7, Phytoreovirus S7 protein; RdRP_4, Viral RNA-dependent RNA-polymerase. (**D**) The pseudoknot structure immediately downstream of the putative frameshift site. The RNA secondary structure was predicted by KnotSeeker program [45] and drawn by VARNAv3-7 program [46]. (**E**) Schematic representation of predicted genome organization of S dsRNA.

*S. sclerotium* strain Sunf-M, which was originally isolated from a sclerotium on a diseased sunflower plant (*Helianthus annuus*), was a normal wild-type strain in colony morphology and virulence. Agarose gel electrophoresis of dsRNA isolated from mycelial extracts of strain Sunf-M revealed the presence of three dsRNA bands, termed L-, M- and S-dsRNA respectively (Figure 1A). The largest L-dsRNA was generally more abundant and migrated slightly slower than the M-dsRNA.

The L-dsRNA was purified from agarose gel with a gel extraction kit, and then subjected to cDNA synthesis, PCR amplification, cloning and sequencing as described before [3]. Computer-assisted sequence assembly showed that the full-length of L-dsRNA cDNA is 9124 bp in length that lacked a poly (A) tail at its 3′-end. Northern blot hybridization confirmed that the sequence was derived from the L-dsRNA (Figure 1B).

Molecular cloning and sequencing of the smallest S-dsRNA was also carried out and the complete nucleotide sequence was determined. Sequencing and Northern hybridization analysis revealed that the S-dsRNA band was actually a doublet consisting of two co-migrating dsRNA segments and thereby the resulting S-dsRNA band was designated as S-1 and S-2 dsRNA, which are 1856 bp and 1783 bp in length, excluding the poly (A) tail, respectively (Figure 1B).

### Characterization of the L-dsRNA

The genomic organization of L-dsRNA revealed that it contains two large open reading frames (ORFs): ORF1 (nt 1089–5006) and ORF2 (nt 5054–9071) in different frames on the genomic plus strand. The L-dsRNA has a long 5′-UTR of 1088 bp, but relatively short 3-UTR of 48 bp (Figure 1C). ORF1 potentially encodes a 1305 amino acid (aa) protein with a predicted molecular mass of 144 kDa. A sequence search with BLASTp showed that it shares significant sequence similarity (E value of ≤ 3e-20) with only the hypothetical proteins of three unclassified dsRNA viruses: Grapevine associated totivirus-2 (GrAV-2) [22], *Fusarium graminearum* dsRNA mycovirus-3 (FgV-3) [23] and *Phlebiopsis gigantea* mycovirus dsRNA 2 (PgRV-2) [24] in the databases. Although a search of conserved domain database (CDD) revealed a significant match (E value of 8.09e-03) with a partial consensus sequence of the phosphoheptose isomerase (SIS_GmhA; cd05006) (Figure 1C), the function of ORF1 protein is unclear.

**Figure 2:**
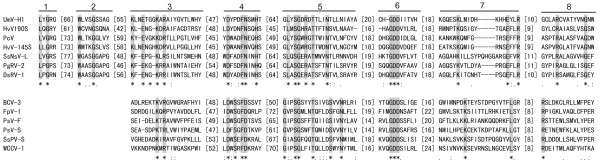
**Comparison of the conserved motifs of RdRps of selected dsRNA mycoviruses including the putative RdRps encoded by SsNsV-L and SsPV-S.** Numbers 1–8 refer to the eight conserved motifs characteristic of RdRps of RNA viruses. The amino acid positions corresponding to conserved motifs 1 and 2 for the RdRps of viruses in the family *Partitiviridae* are not well-defined and, therefore, they are not presented. Asterisks, colons and dots indicate identical amino acid residues (gray shaded), conserved amino acid residues and semi-conserved amino acid residues, respectively. Numbers in square brackets correspond to the number of amino acid residues separating the motifs. See Additional file 2: Table S1 and Additional file 1: Figure S3 for abbreviations of virus names and viral protein accession numbers.

ORF2 potentially encodes a 1338-aa protein with a predicted molecular mass of 146.2 kDa. BLASTp searches showed that this protein was most closely related (E value of ≤ 9e-92) to the putative RdRps of the same three viruses mentioned above as well as another unclassified dsRNA virus *Diplodia scrobiculata* RNA virus 1 (DsRV-1; accession no. NC_013699). In addition, this protein also shares significant sequence similarity with RdRps of members of the families *Totiviridae* and *Chrysoviridae*. CDD searches and multiple protein alignment confirmed that ORF2-encoded protein contained a conserved viral RdRp domain (RdRp_4; pfam02123) with eight conserved motifs characteristic of the RdRps in dsRNA viruses (Figure 2).

Intriguingly, a search of CDD revealed that a region of L-dsRNA ORF2 downstream of the RdRp domain shared significant sequence similarity (E value of 5.96e-03) with a consensus sequence of the Phytoreo_S7 domain (pfam07236, aa 266–349) (Figure 1C). The Phytoreo_S7 domain is characteristic of a family consisting of several phytoreovirus S7 proteins known to be viral core proteins with nucleic acid binding activities. The phytoreovirus S7 domain has also been found in FgV-3 [23], chrysovirus *Penicillium chrysogenum virus* (PcV) [25] as well as other viruses (see below).

The ORF2 is likely expressed via a −1 frameshift mechanism as a fusion protein with ORF1-encoded protein. A heptanucleotide sequence (GAAAAAC_4997-5003_), located in a stretch in-frame with ORF1 stop codon region (nt 4987–5054) and upstream of ORF2 start codon, was identified as a putative shifty heptamer motif that could facilitate ribosomal frameshifting (Figure 1C). In addition, a pseudoknot structure that facilitates pausing of the translating ribosome and increasing the frequency of frameshifting [26,27] was also found immediately downstream of the shifty heptamer (nt 5027–5114) (Figure 1D). Similar genomic organization and expression strategy are characteristic of some members of the family *Totiviridae.* Therefore, the results presented in this section suggest that L-dsRNA probably represented a novel dsRNA mycovirus infecting *S. sclerotiorum* strain Sunf-M. This virus was hence named as *Sclerotinia sclerotiorum* nonsegmented virus L (SsNsV-L).

### Characterization of the S-dsRNA

Both S-1 (1856 bp) and S-2 dsRNA (1783 bp) contain a single ORF on their plus-stranded RNA and share conserved sequences in their 5′-UTRs (Figure 1E and Additional file 1: Figure S1). The S-1 and S-2 dsRNAs potentially encode proteins of 579 and 507 aa, respectively. BLASTp searches showed that the S-1 protein had highest sequence similarity (identity of 47%) to the putative RdRp of *Flammulina velutipes* isometric virus (BAH08700.1) and the S-2 protein shared highest sequence similarity (identity of 25%) with the putative CP of *Rosellinia necatrix* partitivirus 2 (BAK53192.1). Both of these two viruses are members of family *Partitiviridae*. Furthermore, CDD database searches and multiple protein alignment revealed that the S-1 protein contained the consensus motifs of partitivirus RdRps (RNA_dep_RNAP, cd01699) (Figure 2). These results suggest that S-dsRNA is a new member of family *Partitiviridae* infecting *S. sclerotiorum* Sunf-M and was named as *Sclerotinia sclerotiorum* partitivirus S (SsPV-S).

### The diverse monopartite lineages of mycovirus-related dsRNA viruses

**Figure 3:**
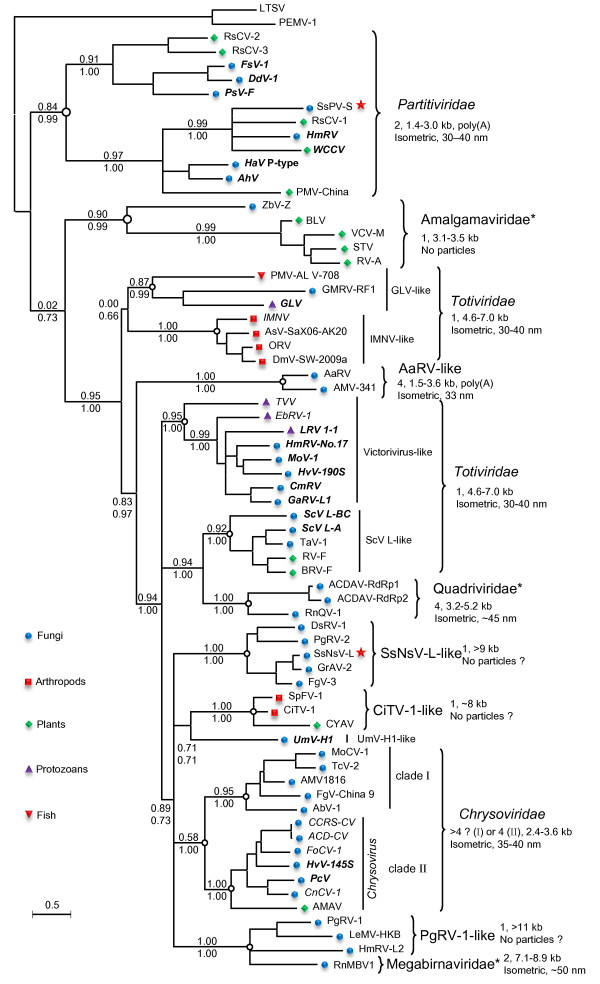
**Phylogenetic tree of mycovirus-related dsRNA viruses.** The tree presented here is the consensus of two trees calculated using phyML-maximum-likelihood (ML) and Bayesian (BI) methods, respectively. Numbers at various nodes indicate, respectively, SH-like approximate likelihood ratio test (SH-aLRT) probabilities (above) and Bayesian posterior probabilities (below). The characteristics (numbers and sizes of genome segments and particle morphology) of different viral lineages are shown. Question mark (?) indicated that characteristics were not determined for all members of this lineage. The viral families that were proposed but have not been recognized by ICTV are indicated by asterisks, and their names are not italicized. The names of the ICTV-recognized or proposed (but not yet recognized) virus species are written in bold italics or italics, respectively. Pentagram indicates the two viruses reported in this study. The host range of viruses was indicated. This tree was rooted with ss(+)RNA viruses. The scale bar corresponds to 0.5 amino acid substitutions per site. See Additional file 2: Table S1 in the supplemental material for abbreviations of virus names and viral protein accession numbers.

**Figure 4:**
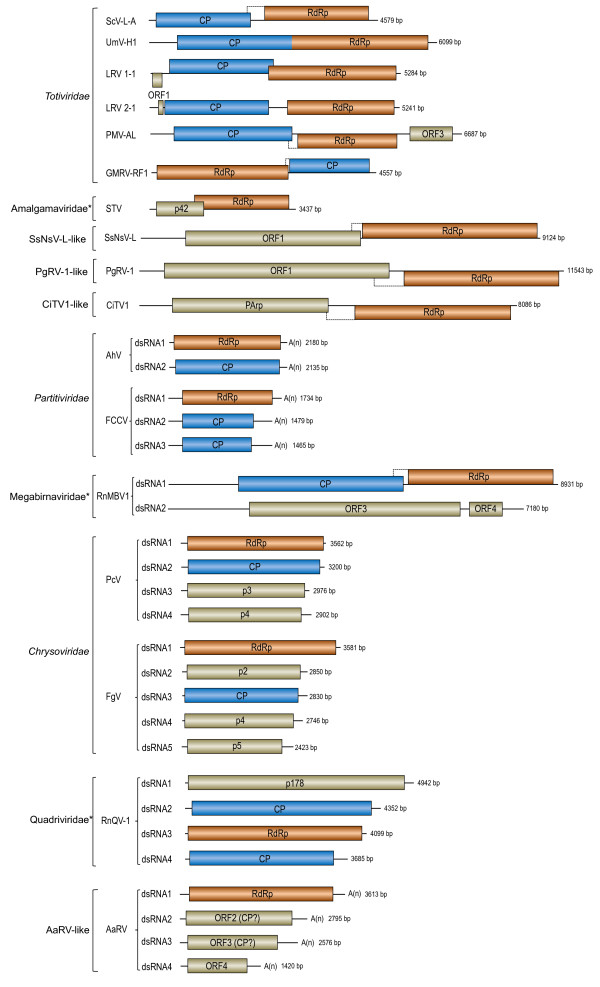
**Genomic organization and comparison of representative viruses in different dsRNA viral lineages.** The Colored boxes and lines represent open reading frames (ORFs) and non-coding sequences, respectively, roughly to scale: orange, RNA-dependent RNA polymerase (RdRp); blue, capsid protein (CP); Brown beige, unknown function. Dotted line boxes indicate possible extension of the downstream ORFs by frameshifting. The viral families that were proposed but have not been recognized by ICTV are indicated by asterisks, and their names are not italicized. See Additional file 2: Table S1 in the supplemental material for abbreviations of virus names.

To elucidate the evolution of mycovirus-related dsRNA viruses including SsNsV-L and SsPV-S, we compared the genome structures and performed phylogenetic analyses for representative members of the families *Chrysoviridae*, *Partitiviridae* and *Totiviridae* as well as other unclassified dsRNA viruses (Figures 3 and 4, Additional file 2: Table S1, and Additional file 1: Figure S2). The analyses revealed that there are diverse evolutionary lineages in the known mycovirus-related dsRNA viruses including at least ten monopartite, three bipartite, one tripartite and three quadripartite lineages.

The monopartite dsRNA family *Totiviridae* is not a monophyletic group since it comprises five distant evolutionary lineages: GLV-like, IMNV-like, victorivirus-like, ScV L-like, and UmV-H1 (Figure 3 and Additional file 1: Figure S2). In addition, there were variations in genome structures for several members compared with typical members (e.g. ScV-L-A) of the family *Totiviridae* (Figure 4). For example, the genome of Piscine myocarditis virus AL V-708 (PMV-AL V-708) contains an extra ORF at its 3′-terminal other than the CP and RdRp ORFs [28] (Figure 4); this is not known to occur in other totiviruses. In addition, contrary to typical totiviruses, the RdRp ORF of Glomus sp. RF1 medium virus (GMRV-RF1) is located at the 5′ terminal half of the genome upstream of CP ORF (Figure 4).

In addition to the five totiviral lineages, the unclassified monopartite dsRNA viruses consist of five other evolutionary lineages: *Amalgamaviridae*, CiTV-1-like, PgRV-1-like, SsNsV-L-like, and NrV-L1, which are genetically distantly related to each other. The Southern tomato virus (STV) and three other related viruses isolated from plants [6-8,29] clustered together and were distantly related to totiviruses. Their genomes are shorter than those of totiviruses and no typical virions are associated with them. A new family, *Amalgamaviridae*, has been proposed to accommodate these four viruses [6]. Our phylogenetic analysis revealed that a yeast virus Zygosaccharomyces bailii virus Z (ZbV-Z) was also likely to be the member of this proposed family. The SsNsV-L-like lineage contained SsNsV-L and four other mycoviruses (Figure 3 and Additional file 1: Figure S2). They have similar genome structures with typical members of the family *Totiviridae* but their genomes (>9 kb) are much larger than those of known totiviruses (Figure 4). It has been reported that the PgRV-2 and FgV-3 possibly do not form true virions [23,24]. It remains to be determined if SsNSV-L has a capsid. The CiTV-1-like lineage included two insect viruses and an unclassified plant virus. Despite the similarity in genome organization with members of the family *Totiviridae*, their genomes (~8 kb) were slightly larger than those of typical totiviruses (4.6–7.0 kb) (Figure 4) and might not assemble in conventional virions [30]. The *P. gigantean* mycovirus dsRNA 1 (PgRV-1)-like lineage consists of three large monopartite dsRNA viruses and no viral particles have been reported in association with infections by PgRV-1 [24]. A mycovirus *Nectria radicicola* virus L1 (NrV-L1) is most closely related to partitiviruses and has been assigned as an unclassified member of the family *Partitiviridae* in database. However, its genome is 6 kb in length [31], clearly suggesting that it is not likely to be a partitivirus. Hence, it may represent a novel group of monopartite dsRNA viruses.

### The diverse bipartite lineages of mycovirus-related dsRNA viruses

The family *Partitiviridae* generally consists of viruses with bipartite genomes comprising two dsRNA segments. All members of this family clustered together in the phylogenetic tree and could be further separated into four clades: clade I-IV (Figure 3 and Additional file 1: Figure S3). The recently isolated mycovirus *Rosellinia necatrix* megabirnavirus 1 (RnMBV1), a member of the proposed family *Megabirnaviridae*[9] was most closely related to the PgRV-1-like monopartite lineage (Figure 3), but it has a bipartite genome encapsidated in isometric virions. Therefore, it may represent a novel bipartite evolutionary lineage of dsRNA viruses. More recently, a novel bipartite dsRNA mycovirus *Botrytis porri* RNA virus 1 (BpRV1) was isolated from *Botrytis porri* that belongs to a separate clade distinct from those of other known RNA mycoviruses [32]. Interestingly, the BpRV1-related bipartite dsRNA virus was also isolated from *S. sclerotiorum* in our lab (Liu LJ et al., unpublished data). These results indicated that there are diverse evolutionary lineages of bipartite dsRNA viruses in nature.

### The tripartite lineage of mycovirus-related dsRNA viruses

Although the typical patitiviruses have two genome segments, some viruses in clade IV harbor three segments, two of which encoded CPs. These CPs formed two sister groups in the CP tree and the two CPs in each virus clustered within different groups (see Additional file 1: Figure S3B), suggesting that a possible duplication event occurred before the divergence of these viruses. Because the genomes of many viruses in the clade IV were not completely sequenced, it is not yet known whether all members of this clade possess two CP genes.

### The diverse quadripartite lineages of mycovirus-related dsRNA viruses

Currently, ICTV recognizes six viruses as members or probable members in the family *Chrysoviridae*[5] (Figure 3). Phylogenetic analysis revealed that other related viruses might also be members of this family (Figure 3 and Additional file 1: Figure S2). The extended family is a monophyletic group and can be further divided into two subgroups: clade I and clade II. Members of clade II have only 4 genome segments whereas members of clade I generally have more than four segments. At present, none of clade I viruses is recognized as member or probable member of the genus *Chrysovirus*. It is worth noting that *Aspergillus* mycovirus 1816 (AMV1816) has been incorrectly assigned by the GenBank as an unclassified member of the family *Totiviridae* in database. Four dsRNA segments have been shown to be associated with this virus [33]. Our phylogenetic analysis also suggests that it is probably a member of *Chrysoviridae*.

Like members of the family *ChrysoviridaeAlternaria alternate* dsRNA mycovirus (AaRV) [34] and *Aspergillus* mycovirus 341 (AMV-341) have four genomic dsRNA segments that are encapsidated in isometric virions. However, each genomic segment has a 3′-poly (A) tail, which is not found in chrysoviruses. In addition, the dsRNA 4 segments of these two viruses (~1.5 kb) are much shorter than those of chrysoviruses (~2.4 kb) (Figure 4). Furthermore, phylogenetic analysis revealed that these two viruses are distantly related to chrysoviruses (Figure 3). These results suggest that the two viruses are members of a novel lineage of quadripartite dsRNA viruses.

Rosellinia necatrix quadrivirus 1 (RnQV1), a novel dsRNA virus, has recently been reported from *Rosellinia necatrix*[35]. Its genome encompasses four segments but all of which are much larger than those of chrysoviruses (Figure 4). Interestingly, the genome of Amasya cherry disease-associated mycovirus (ACDAV) also encompasses four segments but two of which putatively encode RdRps that are related to each other [36] and which cluster with RnQV1 in the phylogenetic tree (Figure 3). It has been suggested that the two RdRps of ACDAV might derive from two related viruses [35]. Although RnQV1 and ACDAV clustered within the totiviral clade (Figure 3), the feature of multipartite genome indicated that they represented a new lineage of quadripartite dsRNA viruses. Based on the properties of RnQV1, a new family *Quadriviridae* has been proposed [35].

### Possible origin of multipartite dsRNA viral lineages

From both phylogenetic trees and network, we can delineate different lineages of multipartite dsRNA viruses: AaRV-like, RnQV1-like, RnMBV1 and chrysoviruses. These are generally most closely related to certain monopartite dsRNA viruses and the monopartite viruses were branching deeply at the base of the multipartite viral lineages. These results suggest that multipartite dsRNA viruses likely evolved from different monopartite dsRNA viruses. The extra segments in monopartite dsRNA viruses could be generated through gene acquisition (e.g. RnMBV1 and chrysoviruses) and/or genome separation (e.g. RnQV1 and AaRV). In addition, phylogenetic analysis places the NrV-L1 at the base of partitiviral clade III (see Additional file 1: Figure S3B), raising the possibility that the ancestor of clade III partitiviruses possibly originated from NrV-L1-like monopartite viruses. This finding raised our awareness of the possibility that the partitiviruses in the three other clades probably also evolved from different monopartite dsRNA viruses that have yet to be discovered. Therefore, generation of multipartite genomes may be an important macroevolutionary mechanism in dsRNA viruses.

### Taxonomy of mycovirus-related dsRNA viruses

In this study, genome comparisons and phylogenetic analyses revealed that at least 10 monopartite, 3 bipartite, 1 tripartite and 3 quadripartite lineages of mycovirus-related dsRNA viruses are known. Among these, some lineages have been considered as members or tentative members of the families, *Totiviridae*, *Partitiviridae* and *Chrysoviridae*. However, the taxonomy of viruses belonging to the other evolutionary lineages has yet to be considered. Because of differences in genome organization, particle morphology and phylogeny from members of these three families, the establishment of new virus families or new genera is warranted to accommodate the unclassified dsRNA viral lineages.

Current taxonomy of genera in the family *Partitiviridae* is based on viral hosts (plants or fungi). Our phylogenetic analysis, however, shown that partitiviral clade I and II consisted of a mosaic of plant partitiviruses (of genus *Alphacryptovirus*) and fungal partitiviruses (of genus *Partitivirus*). Therefore, the host taxon is not a distinguishing character and it does not reflect the true evolutionary relationships of viruses. Because classification of viruses based on phylogeny not only helps to understand the evolution of viruses but also facilitates the prediction of new virus emergence. Therefore, we propose the establishment of four new genera to reflect the four clades in family *Partitiviridae*.

It has been considered that dsRNA viruses are polyphyletic and have originated from different lineages of positive-strand RNA viruses [37-39]. The families *Partitiviridae* and *Totiviridae* have been suggested to belong to the picorna-like superfamily [40]. Our study revealed that members of the family *Chrysoviridae* and other diverse evolutionary lineages of mycovirus-related dsRNA viruses are closely related to totiviruses and partitiviruses. Therefore, these viral lineages may also be included in this expanded superfamily, remarkably expanding the known diversity of the picorna-like viruses.

### Identification of ‘phytoreo S7 domain’

The S7 protein domain is thought to be characteristic of members of the genus *phytoreovirus* in family *Reoviridae*. The finding that S7 domain homologs occur in SsNsV-L, FgV-3 and PcV raised our interest in exploring whether other viruses also have S7 domain homologs. For this purpose, we performed PSI-BLAST searches using the S7 domain sequences of these three viruses and plant phytoreoviruses as seed sequences against NCBI nr database. The results showed that homologs of S7 domain are widely distributed in diverse dsRNA viral lineages (Table 1).

**Table 1:** **Viruses containing homologs of *Phytoreovirus *S7 domain**

**Taxonomy**	**Virus name**	**Abbreviation**	**Aa accession no.**	**Position (aa)**^**a**^
*Reoviridae Phytoreovirus*	Rice gall dwarf virus	RGDV	ABL67643.1	261–359
	Rice dwarf virus	RDV	NP_620530.1	258–362
	Wound tumor virus	WTV	CAA32438.1	259–369
	Tobacco leaf enation phytoreovirus	TLEPV	AAT97064.1	260–370
	Homalodisca vitripennis reovirus	HvReV	YP_002790890.1	264–349
Reoviridae unclassified	Scylla serrata reovirus SZ-2007	SsReV-SZ	ADU86621.1	719–823
*Endornaviridae*	Helicobasidium mompa endornavirus 1	HmEV-1	YP_003280846.1	4681–4776
	Tuber aestivum endornavirus	TaEV	YP_004123950.1	2526–2632
	Gremmeniella abietina type B RNA virus XL1	GaBRV-XL1	YP_529670.1	2761–2862
unclassified monopartite dsRNA viruses	Sclerotinia sclerotiorum nonsegmented virus L	SsNsV-L	JQ513382	893–991
	Fusarium graminearum dsRNA mycovirus-3	FgV-3	YP_003288789.1	866–964
	Diplodia scrobiculata RNA virus 1	DsRV-1	YP_003359178.1	586–680
	Phlebiopsis gigantea mycovirus dsRNA 1	PgRV-1	YP_003541123.1	58–159
	Phlebiopsis gigantea mycovirus dsRNA 2	PgRV-2	CAJ34335.2	838–933
*Totiviridae* unclassified	Glomus sp. RF1 medium virus	GMRV-RF1	BAJ23142.1	11–111
*Chrysoviridae* clade II	Penicillium chrysogenum virus rdrp	PcV_rdrp	YP_392482.1	79–188
	Amasya cherry disease associated chrysovirus rdrp	ACD-CV_rdrp	YP_001531163.1	57–166
	Helminthosporium victoriae 145 S virus rdrp	HvV-145S_rdrp	YP_052858.1	55–164
	Aspergillus fumigatus chrysovirus rdrp	AfCV_rdrp	CAX48749.1	80–185
	Verticillium chrysogenum virus rdrp	VCV_rdrp	ADG21213.1	72–178
	Cherry chlorotic rusty spot associated chrysovirus rdrp	CCRS-CV_rdrp	CAH03664.1	59–166
	Cryphonectria nitschkei chrysovirus 1 rdrp	CnCV-1_rdrp	ACT79258.1	71–177
	Anthurium mosaic-associated virus rdrp	AMAV_rdrp	ACU11563.1	50–161
	Grapevine associated chrysovirus-1 rdrp	GACV-1_rdrp	ADO60926.1	1–105
	Fusarium oxysporum chrysovirus 1 rdrp	FoCV-1_rdrp	ABQ53134.1	1–89
	Amasya cherry disease associated chrysovirus p4	ACD-CV_p4	YP_001531160.1	58–169
	Helminthosporium victoriae 145 S virus p4	HvV-145S_p4	YP_052861.1	77–183
	Cherry chlorotic rusty spot associated chrysovirus p4	CCRS-CV_p4	CAH03667.1	61–169
	Penicillium chrysogenum virus p3	PcV_p3	YP_392484.1	97–206
	Aspergillus fumigatus chrysovirus p3	AfCV_p3	CAX48753.1	91–196
	Verticillium chrysogenum virus p3	VCV_p3	ADG21215.2	57–161
	Cryphonectria nitschkei chrysovirus 1 p4	CnCV-1_p4	ABI20758.1	1–104
*Chrysoviridae* clade I	Tolypocladium cylindrosporum virus 2 rdrp	TcV-2_rdrp	CBY84993.1	47–162
	Magnaporthe oryzae chrysovirus 1 rdrp	MoCV-1_rdrp	YP_003858286.1	43–159
	Fusarium graminearum mycovirus-China 9 rdrp	FgCV rdrp	ADU54123.1	33–153
	Aspergillus mycovirus 1816 rdrp	AMV1816_rdrp	ABX79996.1	1–111
	Agaricus bisporus virus 1 rdrp	AbV-1_rdrp	CAA64144.1	1–95

^a^The positions of Phytoreovirus S7 domain in proteins for alignment and phylogenetic analysis were indicated.

**Figure 5:**
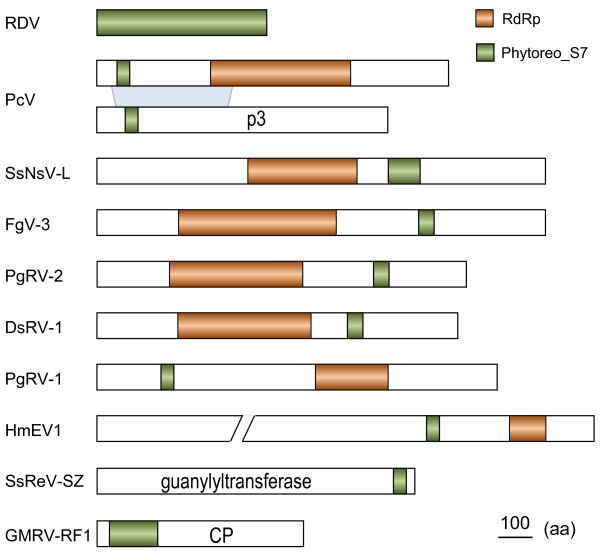
**Locations of Phytoreovirus S7 domain in proteins of different viruses.** See Table 1 for virus names and viral protein accession numbers.

The S7 domain was found in all known chrysoviruses. Interestingly, It occurs in both RdRp and p3 proteins of Clade II chrysoviruses (p3 homolog in several members is p4), but only found in the RdRps of Clade I chrysoviruses. In fact, the 5′-terminal regions of RdRps in Clade II chrysoviruses are homologous with those of their p3/p4 proteins [25] (Figure 5). The S7 domain is located in these 5′-terminal homologous regions. The homology between RdRp and other proteins was not found in Clade I chrysoviruses.

In addition to four members in genus *phytoreovirus*, we also found that the S2 protein (guanylyltransferase) of *Scylla serrata* reovirus SZ-2007 (SsReV-SZ) [41], a member of a proposed new genus in the family *Reoviridae*, contains an S7 domain homolog at its 3′-terminal. This domain was also identified in SsNsV-L-related mycoviruses FgV-3, DsRV-1 and PgRV-2 as well as in another unclassified monopartite dsRNA virus, PgRV-1. Moreover, S7 domain homologs were also found in the CP protein of the totivirus GMRV-RF1 and the polyproteins of three endornaviruses.

**Figure 6:**
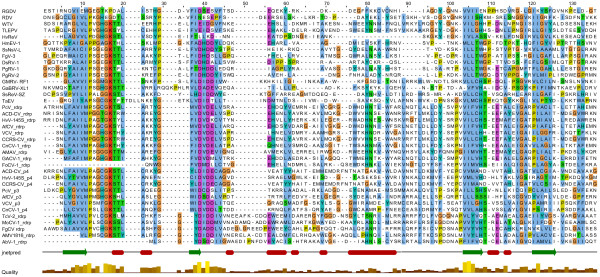
**Multiple alignments of the Phytoreo_S7 domain homologs from diverse viral lineages.** The default color scheme for ClustalW alignment in the Jalview program was used. Jnetpred is the consensus secondary structure prediction: alpha-helices are shown as red rods and beta strands as green arrows. Quality is the quality level for the multiple alignments. See Table 1 for virus names and viral protein accession numbers.

Compared with those of phytoreoviruses, the S7 domain in other viruses contains only partial sequence of the S7 domain. Multiple alignment of the S7 domain revealed that the sequences were likely to be conserved in diverse dsRNA viral lineages (Figure 6). The function of this domain in these non-phytoreoviruses is not known. It will be of interest to determine whether it plays a role in viral RNA binding and packaging.

### Evolution of ‘phytoreo S7 domain’ in diverse viral lineages

Comparison of the domain arrangement in relevant viral proteins shows that the S7 domain is located downstream of the RdRp domain in SsNsV-L, FgV-3, DsRV-1 and PgRV-2. However, the S7 domain resides upstream of the RdRp domain in endornaviruses, chrysoviruses and PgRV-1 (Figure 5). In addition, this domain is also found in other proteins, such as the CP of GMRV-RF1 and the guanylyltransferase of SsReV-SZ. These results suggest that multiple recombination events may have occurred among different viral domains or proteins.

**Figure 7:**
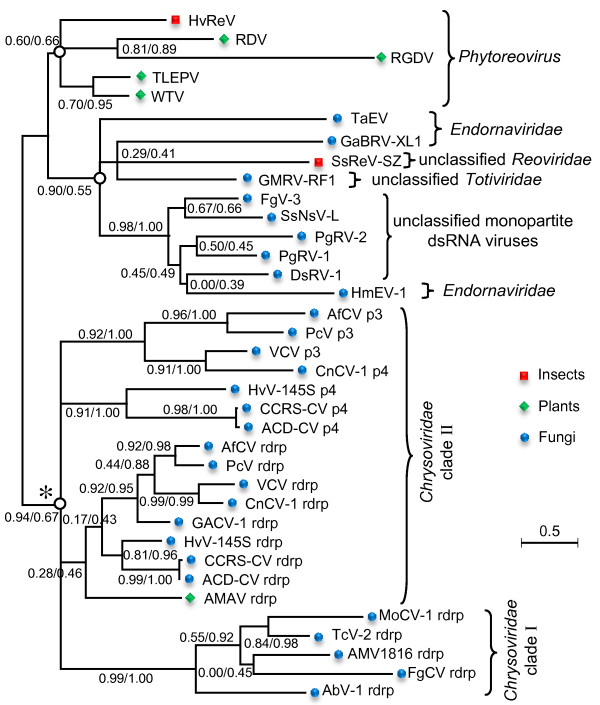
**Phylogenetic tree of the Phytoreo_S7 domain homologs from diverse viral lineages.** This tree is the consensus tree of two trees calculated using phyML-maximum-likelihood (ML) and Bayesian (BI) methods, respectively. Numbers at various nodes indicate, respectively, SH-like approximate likelihood ratio test (aLRT) probabilities/Bayesian posterior probabilities. The topology of asterisk marked clade was evaluated independently. The host range of viruses was indicated. The scale bars correspond to 0.5 amino acid substitutions per site. See Table 1 for virus names and viral protein accession numbers.

**Figure 8:**
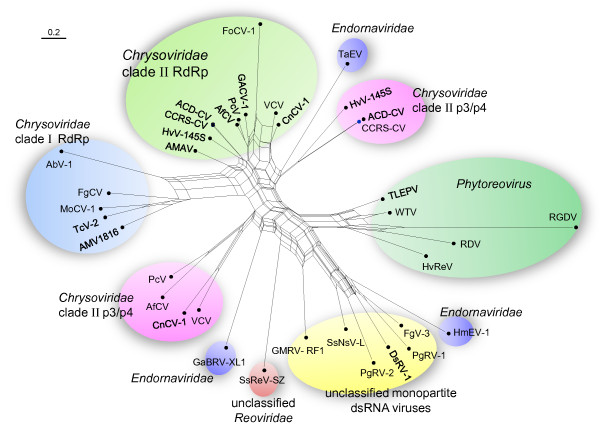
**Neighbor-Net analysis of the Phytoreo_S7 domain homologs from diverse viral lineages.** The analysis was conducted under the WAG model of substitution. Scale bar corresponds to 0.2 amino acid substitutions per site. The major viral lineages are indicated. The box-like appearance in the basal branches of this phylogeny suggests regions of unresolved branches or conflicting phylogenetic signals. See Table 1 for virus names and viral protein accession numbers.

To elucidate the possible evolution of the S7 domain homologs, we constructed phylogenetic tree and network for those from different viral lineages (Figures 7 and 8). The S7 domains from five members of *phytoreovirus* clustered together and formed a distinct clade in both tree and neighbor-net. All of these from chrysoviruses also clustered together. The phylogeny relationships of the p3/p4 of clade II chrysoviruses were consistent with those of their RdRps, suggesting that the ancestor of clade II chrysoviruses also possessed the S7 domain in its RdRp and p3/p4. Considering that the branches of p3/p4 proteins were located at the base of chrysoviruses clade and that the 5′-terminal regions were homologous between RdRps and p3/p4s (Figure 5), it is most likely that the S7 domains were firstly obtained by the p3/p4 proteins of the ancestral clade II chrysoviruses and then transferred to the RdRp proteins by recombination during evolution. Given the close relationships in the neighbor-net (Figure 8), the S7 domains of clade I chrysoviruses may have been acquired by their progenitor from those in RdRps of the ancestor of clade I chrysoviruses via inter-species HGTs.

Some viruses from unrelated families constituted the third clade. The positions of their S7 domains were different (Figure 5). This suggests that HGTs may have occurred among these distant viruses. The closely related four unclassified mycoviruses SsNsV-L, FgV-3, DsRV-1 and PgRV-2 cluster together and the arrangement of their S7 and RdRp domains were also similar, suggesting that the ancestor of these four viruses may have contained the S7 domain. Interestingly, PgRV-1 and PgRV-2 that coinfect one fungal strain clustered together but the positions of their S7 domains were different, suggesting that HGT between these two viruses may have occurred. Similarly, the endornavirus *Helicobasidium mompa* endornavirus 1 (HmEV-1) [35] possibly acquired its S7 domain from the DsRV-1-like virus via HGT.

dsRNA viruses represent an extremely divergent group. Although RdRps are the most highly conserved proteins among RNA viruses, there is little primary-sequence similarity among those encoded by dsRNA viruses from different genera, even those belonging to the same family [1]. For example, the RdRps from several distinct genera in the family *Reoviridae* show only 10–20% amino acid identity [42]. So far, the phylogenetic relationship of members of *Reoviridae* with viruses in other families or genera is not clear. Therefore, the obvious conservation of S7 domain in diverse dsRNA viral lineages even from different families implies that it is not likely to be the results of vertical inheritance. Furthermore, the S7 domain only occurred in certain viruses of a given viral group; their phylogenies were not consistent with those of RdRps. For example, only three members of the family *Endornaviridae* have the S7 domains, and these did not cluster together in S7 domain tree (Figure 6). In addition, the S7 domains in the non-phytoreoviruses are truncated and have different domain arrangement with RdRps (Figure 5). Given these facts, the S7 domain sequences in non-phytoreoviruses were most likely acquired from ancestral phytoreoviruses and then horizontally transferred repeatedly among these diverse dsRNA viral lineages. This suggests that HGT between different virus species is an important macroevolutionary mechanism in dsRNA viruses.

## Conclusions

In summary, we report the molecular properties of two novel mycoviruses from *S. sclerotiorum* strain Sunf-M. One is a monopartite virus representing a distinct evolutionary lineage of dsRNA viruses and the other is a bipartite virus, a new member of the family *Partitiviridae.* Comprehensive phylogenetic analyses and genome comparisons revealed that there are at least ten monopartite, three bipartite, one tripartite and three quadripartite lineages in the known mycovirus-related dsRNA viruses and those multipartite lineages possibly evolved from different monopartite dsRNA viruses. Moreover, we found that homologs of Phytoreo_S7 Domain are widely distributed in diverse non-phytoreovirus lineages, including, chrysoviruses, endornaviruses as well as some unclassified dsRNA mycoviruses. We further provided convincing evidence that multiple HGT events may have occurred among dsRNA viruses from different families. Our study provides an insight into the phylogeny and evolution of dsRNA viruses and reveals that HGT between different viruses and generation of multipartite genomes are important macroevolutionary mechanisms in dsRNA viruses.

## Methods

### Fungal strain and culture conditions

Wild type *S. sclerotiorum* isolate Sunf-M was obtained from sunflower (*Helianthus annuus*) in Hohhot, Inner Mongolia, China. Isolate was routinely cultured on PDA at 20°C and stored at 4°C in PDA tube slants. Small mycelial agar plugs were grown out on cellophane membranes on top of PDA at 20°C for 2 day, and then the mycelium was harvested from the cellophane membranes for dsRNA extraction.

### DsRNA extraction and purification

A previously described procedure for dsRNA extraction [43] was used with minor modifications. Briefly, the mycelium was ground in liquid nitrogen with mortar and pestle. dsRNA was extracted from the mycelium and purified to remove traces of DNA and ssRNA by digestion with S1 nuclease and DNase I (TaKaRa). The dsRNA preparations were fractionated on 1.0% agarose gel and stained with ethidium bromide. The isolated dsRNA was agarose gel purified with an AxyPrep™ DNA Gel Extraction Kit (Axygen).

### cDNA synthesis, molecular cloning and sequencing

cDNA synthesis and molecular cloning of the L-dsRNA was performed as previously described [3]. A single-primer amplification method [44] with minor modifications was used to obtain the full-length clones of S-dsRNA. In brief, a 3′ amino-blocked ligase adaptor (5′-pGCATTGCATCATGATCGATCGAATTCTTTAGTGAGGGTTAATTGCC-NH_2_-3′) was ligated to the 3′-end of the purified S-dsRNA segment using T4 RNA ligase (Fermentas) and the adaptor-ligated dsRNA was then reverse transcribed with a complementary primer 1 (5′-GGCAATTAACCCTCACTAAAG-3′). Following treatment with RNase H (TAKARA), the cDNA strands were annealed for 10 min at 80°C, for 16 h at 65°C and for 3 h at 30°C and the resulting hybrid was filled in with Klenow Fragment (TAKARA). The cDNA was amplified with another complementary primer 2 (5′-TCACTAAAGAATTCGATCGATC-3′). The resulting products were recovered and purified with a gel extraction kit (Axygen), cloned into the pMD18-T vector (TaKaRa). DNA was sequenced by Sanger methods at the Beijing Genomics Institute (BGI). The full genomic sequences of L- and S-dsRNA were completed and confirmed by overlapping of cDNA clones and each base was determined by sequencing at least two independent clones from both orientations. New sequences generated in this study were deposited in GenBank under accession numbers: JQ513382, GQ280377.1 and GQ280378.1.

### Northern blot hybridization

Northern hybridization analysis was performed as previously described [25]. To verify the authenticity of the cDNA clones generated with the purified dsRNA, the cDNA clones from L-dsRNA, S-1 and S-2 dsRNA segments were labeled with ^32^P] dCTP using a radio-labeling kit (TaKaRa) and used to probe the different RNA blots, respectively.

### Data collection and sequence analysis

The genome and protein sequences of the dsRNA viruses used in this study were downloaded from viral genome databases at the NCBI website (http://www.ncbi.nlm.nih.gov/genomes/GenomesHome.cgi?taxid10239). The software package DNAMAN 7 (Lynnon Biosoft, USA) was used for sequence annotations, including nucleotide statistics and ORF searching. Similarity searches of NCBI GenBank database were conducted using the online BLAST program (http://blast.ncbi.nlm.nih.gov/Blast.cgi). Searches for protein domains were performed using NCBI conserved domain database (CDD) (http://www.ncbi.nlm.nih.gov/Structure/cdd/wrpsb.cgi). The pseudoknot structure of L-dsRNA was predicted by the KnotSeeker program [45] and was visualized using program VARNAv3-7 [46].

### Sequence alignment and phylogenetic analysis

Multiple alignments of protein sequences were constructed using the M-Coffee web server (http://tcf_dev.vital-it.ch/apps/tcoffee/play?name=mcoffee), which combines the output of several popular aligners into one single multiple sequence alignments. Phylogenetic trees were estimated using two independent methods: Maximum-Likelihood (ML) and Bayesian inference (BI) on the aligned amino acid sequences. BI trees were constructed with MrBayes-3.1.2 [47],using WAG models of amino acid substitution with invgamma (+I + Γ), performing two runs each of four Monte Carlo Markov Chains (MCMCs), sampling every 1000th iteration over 1.1 × 10^6^ generations after a burn-in of 101 samples. ML trees were inferred with PhyML 3.0 [48], using the best-fit model selected by ProtTest2.4 [49] for each dataset, with SPRs algorithms and 8 categories of γ-distributed substitution rates. The reliability of internal branches was evaluated based on SH-aLRT supports. The resulting BI and ML trees were then used to construct the consensus tree for each alignment using TREE-PUZZLE5.2 [50].

Considering that conflicting phylogenetic signals can lead to tree reconstruction artifacts, we also constructed phylogenetic networks with SplitsTree4 program (http://www.splitstree.org/), using Neighbor-Net method and WAG model of substitution. The unresolved branches or conflicting phylogenetic signals in alignments could be indicated by the box-like structures in neighbor-nets graphs.

## Abbreviations

Aa: Amino acid; aLRT: Approximate likelihood ratio test; BLAST: Basic Local Alignment Search Tool; CDD: Conserved Domain Database; CP: Capsid protein; ds: Double-stranded; HGT: Horizontal gene transfer; MCMCs: Monte Carlo Markov Chains; ML: Maximum-likelihood; NCBI: National Center for Biotechnology Information; ORFs: Open reading frames; RdRp: RNA-dependent RNA polymerase; Ss: Single-stranded.

## Competing interests

The authors declare that they have no competing interests.

## Authors’ contributions

HL, YF and DJ conceived and designed the study; HL performed the computational analyses and lab experiments; HL, YF, DJ, SAG, GL, JX, JC, YP, and XY analyzed data; and HL, YF, DJ and SAG wrote the paper. All authors read and approved the final manuscript.

## Supplementary Material

Additional file 1 **Figures S1–S3.** This file includes 3 supplementary figures. Figure S1 shows the alignments of 5′-untranslated regions (UTRs) of SsPV-S S-1 and S-2 segments. Figure S2 shows Neighbor-Net analysis of mycovirus-related dsRNA viruses. Figure S3 shows the ML phylogenetic trees of the RdRps and CPs of viruses in the family *Partitiviridae.* Click here for file

Additional file 2 **Table S1.** This file contains 1 supplementary Table. Tabe S1 lists the viruses selected for phylogenetic analysis. Click here for file
