# Supplementary material for: Evolutionary genomics of mycovirus-related dsRNA viruses reveals cross-family horizontal gene transfer and evolution of diverse viral lineages
Source: BMC Evol Biol. 2012 Jun 20;12:91. doi: 10.1186/1471-2148-12-91 (PMC3483285; doi:10.1186/1471-2148-12-91)
Supplement: Additional file 1 — Figures S1–S3. This file includes 3 supplementary figures. Figure S1 shows the alignments of 5′-untranslated regions (UTRs) of SsPV-S S-1 and S-2 segments. Figure S2 shows Neighbor-Net analysis of mycovirus-related dsRNA viruses. Figure S3 shows the ML phylogenetic trees of the RdRps and CPs of viruses in the family Partitiviridae. [file 1471-2148-12-91-S1.pdf]

Additional File 1

Supplementary figure legends:

Figure S1 Alignments of 5'-untranslated regions (UTRs) of SsPV-S S-1 and S-2 segments.

The start codon was indicated with red line.

Figure S2 Neighbor-Net analysis of mycovirus-related dsRNA viruses.

The analysis was conducted under the WAG model of substitution. Scale bar corresponds to 1.0 amino acid substitutions per site. The major viral lineages are indicated. The box-like appearance in the basal branches of this phylogeny suggests regions of unresolved branches or conflicting phylogenetic signals. See Table S1 in the supplemental material for abbreviations of virus names and viral protein accession numbers.

Figure S3 ML phylogenetic trees of the RdRps (A) and CPs (B) of viruses in the family *Partitiviridae*.

The GenBank accession numbers of viral proteins are included in taxon names. Viral lineages are colour-coded to reflect their host range. The trees were rooted with STV-like lineages. Only p-values of the SH-like approximate likelihood ratios (SH-aLRT) >0.5 (50%) are indicated. All scale bars correspond to 0.5 amino acid substitutions per site.

```
S-1   1  AGATTAATTATCTTAGTCCACTTTCTAAGTTCAACGAAAATTAAATCAAAAAGC  54
S-2   1  AGATTAATTATCTTAGTCTACTTTCTAAGTTCAACGAAAATCAA-TCAAAAAGC  53

S-1  55  TCTTAACCACACAGATCTTTTAAAACTAACA-----------ATG  88
S-2  54  TTTTAACCACACAAATCTTTTAAAACTAACTCTCTACTCAAGATG  98
                                                  start codon
```

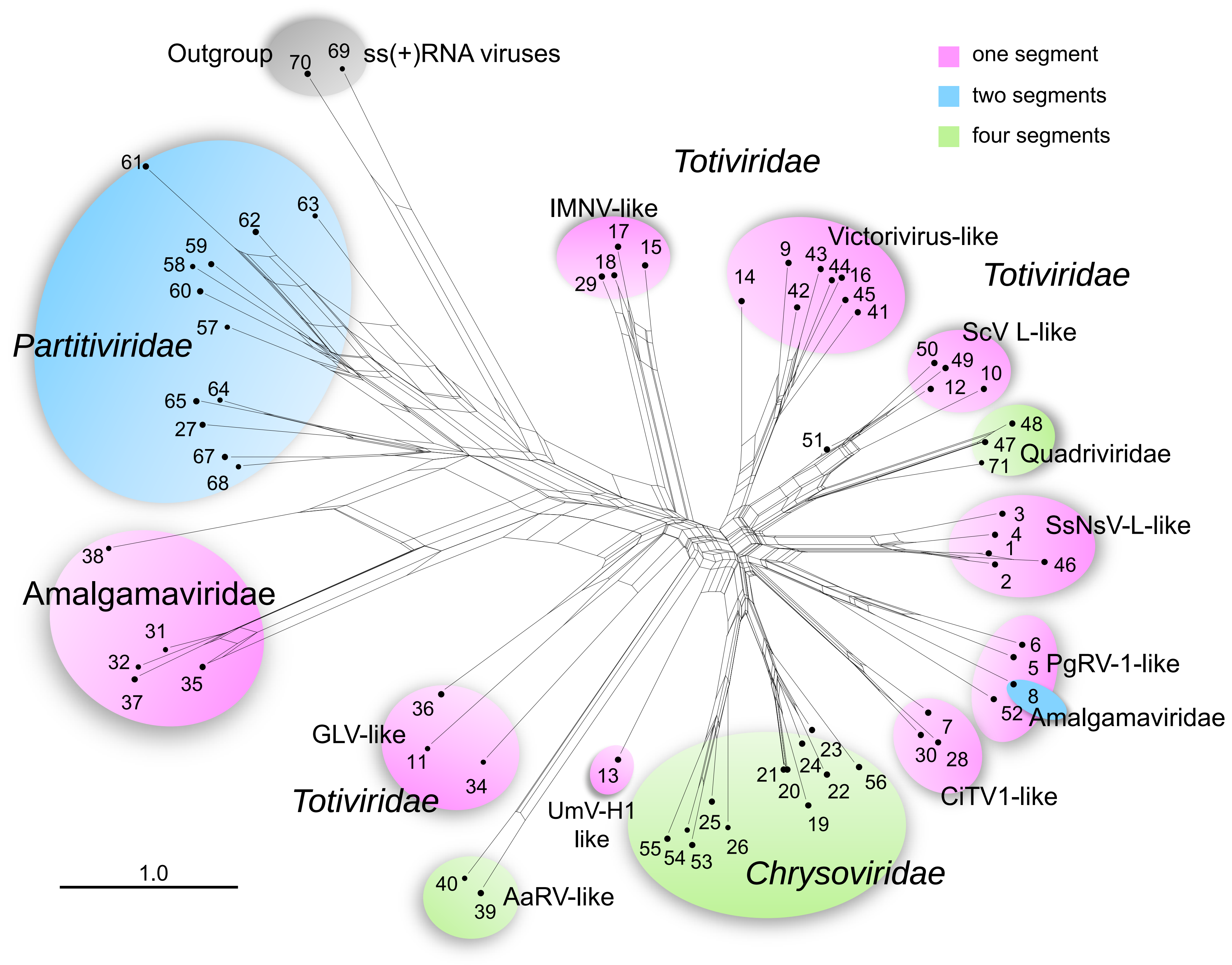
Outgroup
ss(+)RNA viruses
one segment
two segments
four segments
Totiviridae
IMNV-like
Victorivirus-like
Totiviridae
Partitiviridae
ScV L-like
Quadriviridae
SsNsV-L-like
Amalgamaviridae
PgRV-1-like
Amalgamaviridae
GLV-like
Totiviridae
UmV-H1 like
CiTV1-like
Chrysoviridae
AaRV-like
1.0

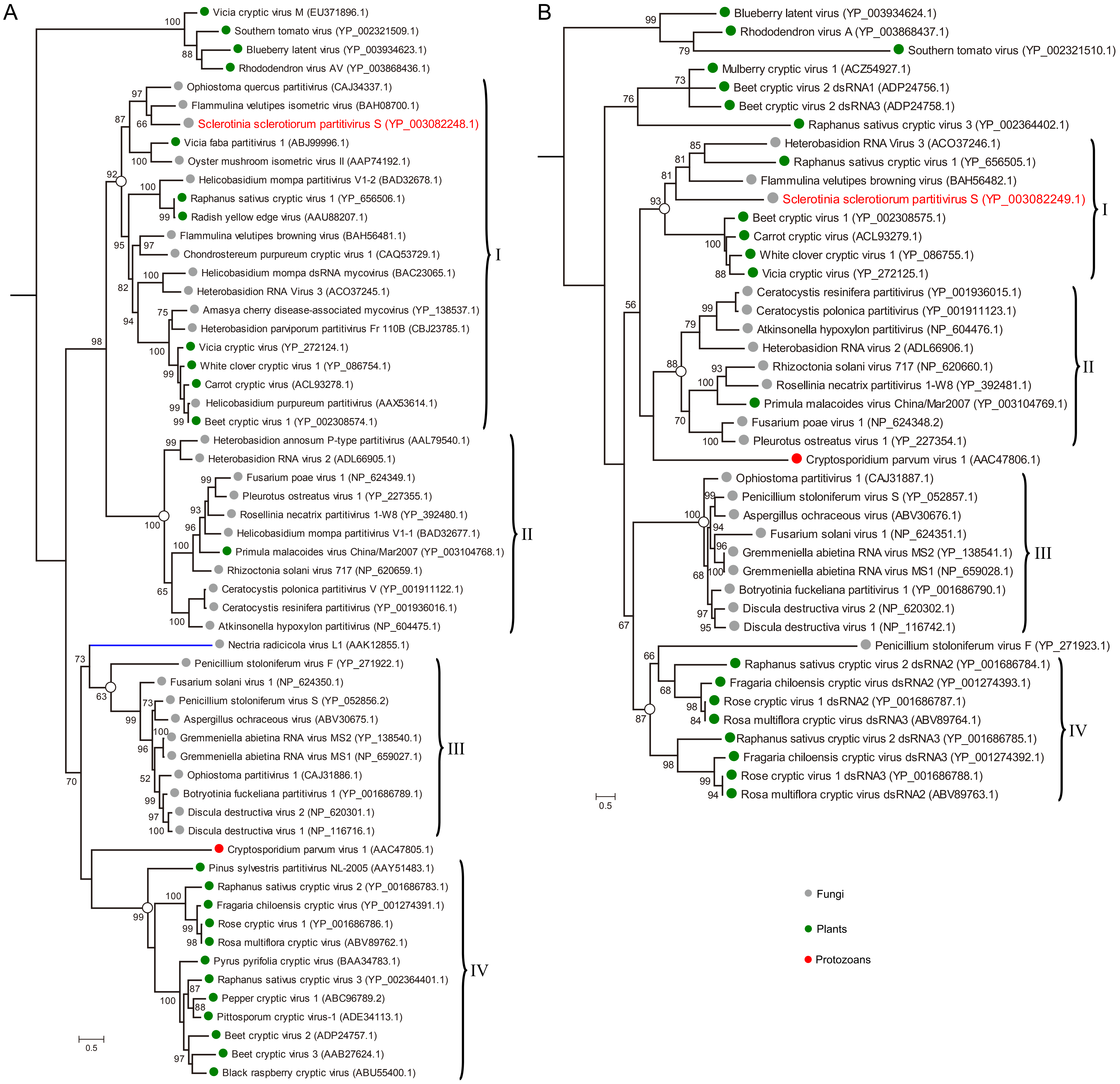
A
Vicia cryptic virus M (EU371896.1)
Southern tomato virus (YP_002321509.1)
Blueberry latent virus (YP_003934623.1)
Rhododendron virus AV (YP_003868436.1)
Ophiostoma quercus partitivirus (CAJ34337.1)
Flammulina velutipes isometric virus (BAH08700.1)
Sclerotinia sclerotiorum partitivirus S (YP_003082248.1)
Vicia faba partitivirus 1 (ABJ99996.1)
Oyster mushroom isometric virus II (AAP74192.1)
Helicobasidium mompa partitivirus V1-2 (BAD32678.1)
Raphanus sativus cryptic virus 1 (YP_656506.1)
Radish yellow edge virus (AAU88207.1)
Flammulina velutipes browning virus (BAH56481.1)
Chondrostereum purpureum cryptic virus 1 (CAQ53729.1)
Helicobasidium mompa dsRNA mycovirus (BAC23065.1)
Heterobasidion RNA Virus 3 (ACO37245.1)
Amasya cherry disease-associated mycovirus (YP_138537.1)
Heterobasidion parviporum partitivirus Fr 110B (CBJ23785.1)
Vicia cryptic virus (YP_272124.1)
White clover cryptic virus 1 (YP_086754.1)
Carrot cryptic virus (ACL93278.1)
Helicobasidium purpureum partitivirus (AAX53614.1)
Beet cryptic virus 1 (YP_002308574.1)
I
Heterobasidion annosum P-type partitivirus (AAL79540.1)
Heterobasidion RNA virus 2 (ADL66905.1)
Fusarium poae virus 1 (NP_624349.1)
Pleurotus ostreatus virus 1 (YP_227355.1)
Rosellinia necatrix partitivirus 1-W8 (YP_392480.1)
Helicobasidium mompa partitivirus V1-1 (BAD32677.1)
Primula malacoides virus China/Mar2007 (YP_003104768.1)
Rhizoctonia solani virus 717 (NP_620659.1)
Ceratocystis polonica partitivirus V (YP_001911122.1)
Ceratocystis resinifera partitivirus (YP_001936016.1)
Atkinsonella hypoxylon partitivirus (NP_604475.1)
II
Nectria radicicola virus L1 (AAK12855.1)
Penicillium stoloniferum virus F (YP_271922.1)
Fusarium solani virus 1 (NP_624350.1)
Penicillium stoloniferum virus S (YP_052856.2)
Aspergillus ochraceous virus (ABV30675.1)
Gremmeniella abietina RNA virus MS2 (YP_138540.1)
Gremmeniella abietina RNA virus MS1 (NP_659027.1)
Ophiostoma partitivirus 1 (CAJ31886.1)
Botryotinia fuckeliana partitivirus 1 (YP_001686789.1)
Discula destructiva virus 2 (NP_620301.1)
Discula destructiva virus 1 (NP_116716.1)
III
Cryptosporidium parvum virus 1 (AAC47805.1)
Pinus sylvestris partitivirus NL-2005 (AAY51483.1)
Raphanus sativus cryptic virus 2 (YP_001686783.1)
Fragaria chiloensis cryptic virus (YP_001274391.1)
Rose cryptic virus 1 (YP_001686786.1)
Rosa multiflora cryptic virus (ABV89762.1)
Pyrus pyrifolia cryptic virus (BAA34783.1)
Raphanus sativus cryptic virus 3 (YP_002364401.1)
Pepper cryptic virus 1 (ABC96789.2)
Pittosporum cryptic virus-1 (ADE34113.1)
Beet cryptic virus 2 (ADP24757.1)
Beet cryptic virus 3 (AAB27624.1)
Black raspberry cryptic virus (ABU55400.1)
IV
0.5
B
Blueberry latent virus (YP_003934624.1)
Rhododendron virus A (YP_003868437.1)
Southern tomato virus (YP_002321510.1)
Mulberry cryptic virus 1 (ACZ54927.1)
Beet cryptic virus 2 dsRNA1 (ADP24756.1)
Beet cryptic virus 2 dsRNA3 (ADP24758.1)
Raphanus sativus cryptic virus 3 (YP_002364402.1)
Heterobasidion RNA Virus 3 (ACO37246.1)
Raphanus sativus cryptic virus 1 (YP_656505.1)
Flammulina velutipes browning virus (BAH56482.1)
Sclerotinia sclerotiorum partitivirus S (YP_003082249.1)
Beet cryptic virus 1 (YP_002308575.1)
Carrot cryptic virus (ACL93279.1)
White clover cryptic virus 1 (YP_086755.1)
Vicia cryptic virus (YP_272125.1)
I
Ceratocystis resinifera partitivirus (YP_001936015.1)
Ceratocystis polonica partitivirus (YP_001911123.1)
Atkinsonella hypoxylon partitivirus (NP_604476.1)
Heterobasidion RNA virus 2 (ADL66906.1)
Rhizoctonia solani virus 717 (NP_620660.1)
Rosellinia necatrix partitivirus 1-W8 (YP_392481.1)
Primula malacoides virus China/Mar2007 (YP_003104769.1)
Fusarium poae virus 1 (NP_624348.2)
Pleurotus ostreatus virus 1 (YP_227354.1)
II
Cryptosporidium parvum virus 1 (AAC47806.1)
Ophiostoma partitivirus 1 (CAJ31887.1)
Penicillium stoloniferum virus S (YP_052857.1)
Aspergillus ochraceous virus (ABV30676.1)
Fusarium solani virus 1 (NP_624351.1)
Gremmeniella abietina RNA virus MS2 (YP_138541.1)
Gremmeniella abietina RNA virus MS1 (NP_659028.1)
Botryotinia fuckeliana partitivirus 1 (YP_001686790.1)
Discula destructiva virus 2 (NP_620302.1)
Discula destructiva virus 1 (NP_116742.1)
III
Penicillium stoloniferum virus F (YP_271923.1)
Raphanus sativus cryptic virus 2 dsRNA2 (YP_001686784.1)
Fragaria chiloensis cryptic virus dsRNA2 (YP_001274393.1)
Rose cryptic virus 1 dsRNA2 (YP_001686787.1)
Rosa multiflora cryptic virus dsRNA3 (ABV89764.1)
Raphanus sativus cryptic virus 2 dsRNA3 (YP_001686785.1)
Fragaria chiloensis cryptic virus dsRNA3 (YP_001274392.1)
Rose cryptic virus 1 dsRNA3 (YP_001686788.1)
Rosa multiflora cryptic virus dsRNA2 (ABV89763.1)
IV
0.5
Fungi
Plants
Protozoans
