# Supplementary material for: Evolutionary genomics of mycovirus-related dsRNA viruses reveals cross-family horizontal gene transfer and evolution of diverse viral lineages
Source: BMC Evol Biol. 2012 Jun 20;12:91. doi: 10.1186/1471-2148-12-91 (PMC3483285; doi:10.1186/1471-2148-12-91)
Supplement: Additional file 2 — Table S1. This file contains 1 supplementary Table. Tabe S1 lists the viruses selected for phylogenetic analysis. [file 1471-2148-12-91-S2.pdf]

Additional File 2

Supplementary Table S1. Viruses selected for phylogenetic analysis.

Number [a)]	Virus name [b)]	Abbreviation	Aa accession no.
1	Sclerotinia sclerotiorum nonsegmented virus L	SsNsV-L	JQ513382
2	Fusarium graminearum dsRNA mycovirus-3	FgV-3	YP_003288789.1
3	Diplodia scrobiculata RNA virus 1	DsRV-1	YP_003359178.1
4	Phlebiopsis gigantea mycovirus dsRNA 2	PgRV-2	CAJ34335.2
5	Phlebiopsis gigantea mycovirus dsRNA 1	PgRV-1	YP_003541123.1
6	Lentinula edodes mycovirus HKB	LeMV-HKB	BAG71788.1
7	Cucurbit yellows-associated virus	CYAV	CAA63099.2
8	Rosellinia necatrix megabirnavirus 1/W779	RnMBV1	YP_003288763.1
9	***Leishmania RNA virus 1-1***	LRV 1-1	NP_041191.1
10	***Saccharomyces cerevisiae virus L-BC [La]***	ScV L-BC	NP_042581.1
11	***Giardia lamblia virus***	GLV	NP_620070.1
12	***Saccharomyces cerevisiae virus L-A [L1]***	ScV L-A	NP_620495.1
13	***Ustilago maydis virus H1***	UmV-H1	NP_620728.1
14	*Trichomonas vaginalis virus*	TVV	NP_620730.2
15	*Penaeid shrimp infectious myonecrosis virus*	IMNV	YP_529549.1
16	***Helminthosporium victoriae virus 190S***	HvV-190S	NP_619670.2
17	Armigeres subalbatus virus SaX06-AK20	AsV-SaX06-AK20	ACH85916.1
18	Drosophila melanogaster totivirus SW-2009a	DmV-SW-2009a	YP_003289293.1
19	***Helminthosporium victoriae 145S virus***	HvV-145S	YP_052858.1
20	*Cherry chlorotic rusty spot associated chrysovirus*	CCRS-CV	CAH03664.1
21	*Amasya cherry disease associated chrysovirus*	ACD-CV	YP_001531163.1
22	***Penicillium chrysogenum virus***	PcV	YP_392482.1
23	*Cryphonectria nitschkei chrysovirus 1*	CnCV-1	ACT79256.1
24	*Fusarium oxysporum chrysovirus 1*	FoCV-1	ABQ53134.1
25	Aspergillus mycovirus 1816	AMV1816	ABX79996.1
26	Agaricus bisporus virus 1	AbV-1	CAA64144.1
27	***Penicillium stoloniferum virus F***	PsV-F	YP_271922.1
28	Spissistilus festinus virus 1	SpFV-1	YP_003800001.1
29	Omono River virus	ORV	BAJ21513.1
30	Circulifer tenellus virus 1	CiTV-1	YP_003800003.1
31	Vicia cryptic virus M	VCV-M	EU371896.1
32	Rhododendron virus A	RV-A	YP_003868436.1
34	Piscine myocarditis virus AL V-708	PMV-AL V-708	ADP37187.1
35	Southern tomato virus	STV	YP_002321509.1
36	Glomus sp. RF1 medium virus	GMRV- RF1	BAJ23141.1
37	Blueberry latent virus	BLV	YP_003934623.1
38	Zygosaccharomyces bailii virus Z	ZbV-Z	NP_624325.1
39	Alternaria alternata dsRNA mycovirus	AaRV	YP_001976142.1

40	Aspergillus mycovirus 341	AMV-341	ABX79997.1
41	*Eimeria brunetti RNA virus 1*	EbRV-1	NP_108651.1
42	***Gremmeniella abietina RNA virus L1***	GaRV-L1	NP_624332.2
43	***Coniothyrium minitans RNA virus***	CmRV	YP_392467.1
44	***Magnaporthe oryzae virus 1***	MoV-1	YP_122352.1
45	***Helicobasidium mompa No.17 dsRNA virus***	HmRV-No.17	NP_898833.1
46	Grapevine associated totivirus-2	GrAV-2	ADO60933.1
47	Amasya cherry disease-associated mycovirus RdRp2	ACDAV-RdRp2	CAJ29959.1
48	Amasya cherry disease-associated mycovirus RdRp1	ACDAV-RdRp1	CAJ29958.1
49	Tuber aestivum virus 1	TaV-1	ADQ54106.1
50	Black raspberry virus F	BRV-F	YP_001497151.1
51	Ribes virus F	RV-F	ACA61232.1
52	Helicobasidium mompa V670 L2-dsRNA virus	HmRV-L2	AB275288.1
53	Magnaporthe oryzae chrysovirus 1	MoCV-1	YP_003858286.1
54	Tolypocladium cylindrosporum virus 2	TcV-2	CBY84993.1
55	Fusarium graminearum mycovirus-China 9	FgV-China 9	ADU54123.1
56	Anthurium mosaic-associated virus	AMAV	ACU11563.1
57	Sclerotinia sclerotiorum partitivirus S	SsPV-S	YP_003082248.1
58	Raphanus sativus cryptic virus 1	RsCV-1	YP_656506.1
59	***Helicobasidium mompa dsRNA mycovirus***	HmRV	BAC23065.1
60	***White clover cryptic virus 1***	WCCV	YP_086754.1
61	***Heterobasidion annosum P-type partitivirus***	HaV P-type	AAL79540.1
62	***Atkinsonella hypoxylon partitivirus***	AhV	NP_604475.1
63	Primula malacoides virus China/Mar2007	PMV-China	YP_003104768.1
64	***Fusarium solani virus 1***	FsV-1	NP_624350.1
65	***Discula destructiva virus 1***	DdV-1	NP_116716.1
67	Raphanus sativus cryptic virus 2	RsCV-2	YP_001686783.1
68	Raphanus sativus cryptic virus 3	RsCV-3	YP_002364401.1
69	***Lucerne transient streak virus***	LTSV	NP_736596.1
70	***Pea enation mosaic virus-1***	PEMV-1	NP_620026.1
71	Rosellinia necatrix quadrivirus 1	RnQV-1	YP_005097975

[a] The numbers are corresponding to Fig S2 in the supplemental material.

[b] The names of the ICTV-recognized or proposed (but not yet officially recognized) virus species are written in Bold italics or italics, respectively.
